# Benchmark Study on the Calculation of ^207^Pb NMR Chemical Shifts

**DOI:** 10.1021/acs.inorgchem.3c04539

**Published:** 2024-03-06

**Authors:** Thomas Gasevic, Julius B. Kleine Büning, Stefan Grimme, Markus Bursch

**Affiliations:** † Mulliken Center for Theoretical Chemistry, Clausius Institute for Physical and Theoretical Chemistry, University of Bonn, Beringstr. 4, 53115 Bonn, Germany; ‡ Max-Planck-Institut für Kohlenforschung, Kaiser-Wilhelm-Platz 1, 45470 Mülheim an der Ruhr, Germany

## Abstract

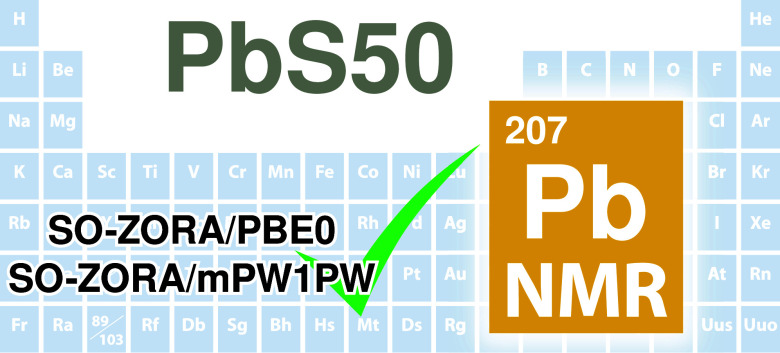

A benchmark set for the computation of ^207^Pb nuclear magnetic resonance (NMR) chemical shifts is presented. The *PbS50* set includes conformer ensembles of 50 lead-containing molecular compounds and their experimentally measured ^207^Pb NMR chemical shifts. Various bonding motifs at the Pb center with up to seven bonding partners are included. Six different solvents were used in the measurements. The respective shifts lie in the range between +10745 and −5030 ppm. Several calculation settings are assessed by evaluating computed ^207^Pb NMR shifts for the use with different density functional approximations (DFAs), relativistic approaches, treatment of the conformational space, and levels for geometry optimization. Relativistic effects were included explicitly with the zeroth order regular approximation (ZORA), for which only the spin–orbit variant was able to yield reliable results. In total, seven GGAs and three hybrid DFAs were tested. Hybrid DFAs significantly outperform GGAs. The most accurate DFAs are mPW1PW with a mean absolute deviation (MAD) of 429 ppm and PBE0 with an MAD of 446 ppm. Conformational influences are small as most compounds are rigid, but more flexible structures still benefit from Boltzmann averaging. Including explicit relativistic treatments such as SO-ZORA in the geometry optimization does not show any significant improvement over the use of effective core potentials (ECPs).

## Abstract 2

The *PbS50* benchmark set for the computation of ^207^Pb NMR chemical shifts is presented. It includes 50 lead-containing molecules with their respective ^207^Pb NMR shifts experimentally measured in solution. Various bonding motifs at the Pb center with up to seven bonding partners are included. The respective shifts lie in a range between +10745 and −5030 ppm. Several calculation settings are assessed by evaluating different density functional approximations, relativistic approaches, treatment of the conformational space, and levels for geometry optimization.

## Introduction

One of the leading analytical tools for the elucidation of chemical structures is nuclear magnetic resonance (NMR) spectroscopy. Respective measurements are commonly applied to NMR-active ^1^H and ^13^C nuclei. Nevertheless, heteronuclei such as ^207^Pb can also reveal toxicity in organisms^1^ and give a valuable insight into the chemical environment around the respective nucleus, e.g., the coordination sphere.^1,2^ Recorded NMR spectra and the NMR measurement itself can already be extremely complicated for small but complex structures. Further, NMR signals for heavier nuclei can occur over a broad range of shifts, which requires time-consuming measurements in order to locate the actual signal. Therefore, theoretical prediction methods have become indispensable for the setup and evaluation of experimental NMR data. Density functional theory (DFT) in combination with gauge-including atomic orbitals (GIAO)^3−6^ is routinely applied to compute NMR chemical shifts, especially because of the excellent compromise between computational cost and accuracy.^7−9^ Although reliable results can be achieved with this approach, it can be difficult to find the right density functional approximation (DFA) among a plethora of DFAs. Therefore, benchmark studies are essential for the choice of DFA. Computations of ^207^Pb NMR shifts have already been carried out for, e.g., dirhodioplumbole,^10^ organolead^11^ and halogen lead complexes,^12^ diarylplumbylenes,^13^ plumbacyclopentadienylidenes,^14^ and endohedral plumbaspherenes.^15^ Further studies include the computation of solid-state ^207^Pb NMR chemical shifts that already reveal the importance of Fock exchange and spin–orbit (SO) effects.^16−20^ Still, they all lack a thorough assessment of the underlying theoretical method based on a diverse set of lead-containing compounds that were measured in solution.^10−15,21^ Due to the presence of unoccupied p-orbitals in group 14 elements, the ^207^Pb nucleus has a huge SO heavy atom effect on the shielding of neighboring light atoms (HALA effects), especially in Pb^II^ compounds.^22^ Heavy atoms in the vicinity of the Pb nucleus can also have a heavy atom effect on the heavy atom Pb (HAHA). Both phenomena require the explicit treatment of relativistic effects, which can be taken into account by the Douglas–Kroll–Hess,^23,24^ the zeroth-order regular approximation (ZORA),^25,26^ or the exact two-component (X2C) approaches.^27−31^ All of them apply a scalar relativistic (SR) treatment but can optionally also include SO coupling, which is necessary to describe the HALA and HAHA effects. As a follow-up to our ^29^Si^32^ and ^119^Sn NMR^33^ chemical shift benchmark sets, we now introduce a set of compounds for the quantum chemical computation of ^207^Pb NMR chemical shifts. The *PbS50* set features 50 unique experimental ^207^Pb NMR chemical shifts of molecular compounds in solution. It includes diverse structures in different chemical environments to cover a broad range of available chemical shifts and represent a large part of the Pb-containing molecular chemical space. The main goal is to find recommendations for the computational setup for predictions of ^207^Pb NMR chemical shifts.

## Computational Details

All quantum chemical computations have been carried out with the xtb 6.4.1,^34,35^ TURBOMOLE 7.6,^36−38^ and AMS 2023.103^39,40^ program packages. Initial geometries were obtained from X-ray crystal structures. If no crystal structure was available, the compounds were manually constructed from similar crystal structures (compounds **39**, **41**, **42**, **44**, and **50**). All structures were preoptimized with the semiempirical quantum mechanical (SQM) GFN2-xTB^41^ method in combination with the ALPB implicit solvation model^42^ for the respective solvent that was used in the NMR experiment. Conformer ensembles (CE) were created with the conformer-rotamer ensemble sampling tool CREST 2.11.3^43,44^ at the GFN2-xTB level of theory. Due to optimization issues, the CEs of compounds **1**, **10**, **20**, **21**, and **34** were computed at the generic force field GFN-FF^45^ level of theory. The ensembles were further refined with the command-line energetic sorting tool CENSO 1.2.0,^46,47^ and still identical conformers were manually removed by inspecting each ensemble. Within the CENSO algorithm, geometries were optimized with the efficient r^2^SCAN-3c^48^ composite DFT method and the COSMO^49^ implicit solvation model in TURBOMOLE. A fine radial integration grid was applied (*radsize* = 10). Solvation free energies were computed with COSMO-RS^50,51^ using the COSMOtherm 19.0^52^ program package (*G*_solv_ option, BP_TZVP_C30_1601 parameterization, *T* = 298.15 K, *p* = 1 atm). Thermostatistical contributions were obtained with the single-point Hessian method^53^ within the modified rigid-rotor harmonic oscillator^54^ approach using GFN2-xTB. All structures were verified as minima on the potential energy surface by the absence of significant imaginary frequencies below −*i*ω = 30 cm^–1^ in the numerical harmonic frequency calculation. The resolution of identity approximation for Coulomb integrals (RI-J) was applied in all calculations carried out with matching auxiliary basis sets in TURBOMOLE.

All ^207^Pb NMR chemical shieldings were computed in ADF from the AMS program package with GIAOs and the COSMO solvation model^55^ for the respective solvent applied in the experimental NMR measurement. Relativistic effects were treated by the SR or the SO variant of ZORA.^25,56^ The density functionals listed in Table 1 were applied in combination with the ZORA/TZP^57^ basis set. The *symmetry* option was turned off, and the *NumericalQuality* was set to good in all computations.

**Table 1:** List of All Tested DFAs and Relativistic Treatments (SR = Scalar Relativistic, SO = Spin–Orbit)^a^

		ZORA	
class	DFA	SR	SO
GGA	PBE^71^	+	+
	revPBE^72^	+	+
	BLYP^73,74^	+	+
	OLYP^75^	+	+
	BP86^76,77^	+	+
	mPW^78^	+	+
	KT2^79^	+	+
hybrid	PBE0^80^	+	+
	B3LYP^81−83^	+	+
	mPW1PW^78^	+	+

a Each DFA was applied with the ZORA/TZP basis set.

For a subset consisting of compounds **26**, **28**–**43**, and **48**, the SO contribution was found to be below 200 ppm. ^207^Pb NMR chemical shieldings for this subset were computed in the ORCA 5.0.4 program package^58,59^ with the CPCM solvation model^60^ for the respective solvent applied in the experimental NMR measurement. SR-ZORA was applied in all calculations together with the ZORA-def2-TZVP basis set^61^ for all elements with *Z* ≤ 36 and SARC-ZORA-TZVP^62,63^ for all with *Z* > 36. The chain-of-spheres approach to RIJCOSX and the Dobson ansatz for τ in meta-GGAs were used throughout. The density functionals PBE, TPSS,^64^ r^2^SCAN,^65^ B97M-V,^66^ TPSSh,^67^ r^2^SCAN0,^68^ ωB97X-V,^69^ and ωB97M-V^70^ were tested in this study.

NMR chemical shifts δ were obtained by referencing the respective shielding constant of compound k to the reference compound PbMe_4_ according to eq 1
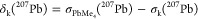
1

To obtain thermally averaged NMR chemical shifts, Boltzmann weights *w*_*i*_ of each conformer *i* in the CE were calculated according to
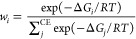
2with the difference in Gibbs free energy between each conformer and the lowest conformer of the ensemble Δ*G*_*i*_ = *G*_*i*_ – *G*_lowest_, the molar gas constant *R*, and the temperature *T* = 298.15 K.

To investigate the influence of the applied method in the geometry optimization, the lowest-lying conformer of each ensemble was reoptimized with the SQM methods GFN1- and GFN2-xTB, the GFN-FF force field, as well as with the SR- and SO-ZORA variants of r^2^SCAN-3c(STO),^84^ as implemented in ADF. Solvation effects were treated with the ALPB and COSMO solvation models, respectively. The *NumericalQuality* was set to good in ADF.

If not stated otherwise, default settings were applied.

## Results and Discussion

### General Considerations

Although quantum chemical calculations of NMR chemical shifts are routinely used, one faces various obstacles in the computational workflow. For example, the question of conformational influences arises when the system under investigation is flexible. Since the measured nucleus is differently shielded in each conformer, it is crucial to consider the whole CE and take the respective populations into account to predict NMR shifts more accurately. Further, the choice of the density functional, basis set, solvation model, and relativistic treatment has to be made for the geometry optimization and the subsequent NMR chemical shielding calculation. For a more general discussion of those effects, see ref (85).

Some of the mentioned problems were previously assessed. For the choice of the basis set, a triple-ζ quality is already sufficient as it is converged with respect to the NMR chemical shift.^32^ The influence of different implicit solvation models has also been proven to be small and is hence not tested here.^86^ Still, explicit solvation effects can become crucial if a polar solvent, such as DMSO or THF, closely coordinates to the solute. This has already been reported, e.g., for ^195^Pt NMR shifts in the works by Autschbach et al.^20,87^ of systems with water molecules explicitly coordinating to the platinum center. Due to the bulky ligands in the investigated compounds, the effect of explicit solvation is expected to be small for this test set. Studies on light-atom NMR shift calculations further investigated more density functionals^88^ than presented in this work. The main reasons for this are the availability and limitations of quantum chemical codes that support relativistic effects beyond scalar. The AMS program package that was used in this work, e.g., does not support meta-GGAs for the computation of NMR shifts. Other program packages to be mentioned here are TURBOMOLE, which features a 2-component implementation at the X2C level^89^ and DIRAC^90^ as well as ReSpect^91^ which both feature a 4-component implementation.

In this work, we study the performance of various density functionals applied for the ^207^Pb NMR chemical shift calculations as well as the influence of the conformational space and relativistic effects on that property. Further, we investigate different levels of theory for the geometry optimization. Finally, recommendations are given for the workflow of predicting ^207^Pb NMR chemical shifts with DFT by using a multilevel approach.

### Benchmark Set

**Figure 1:**
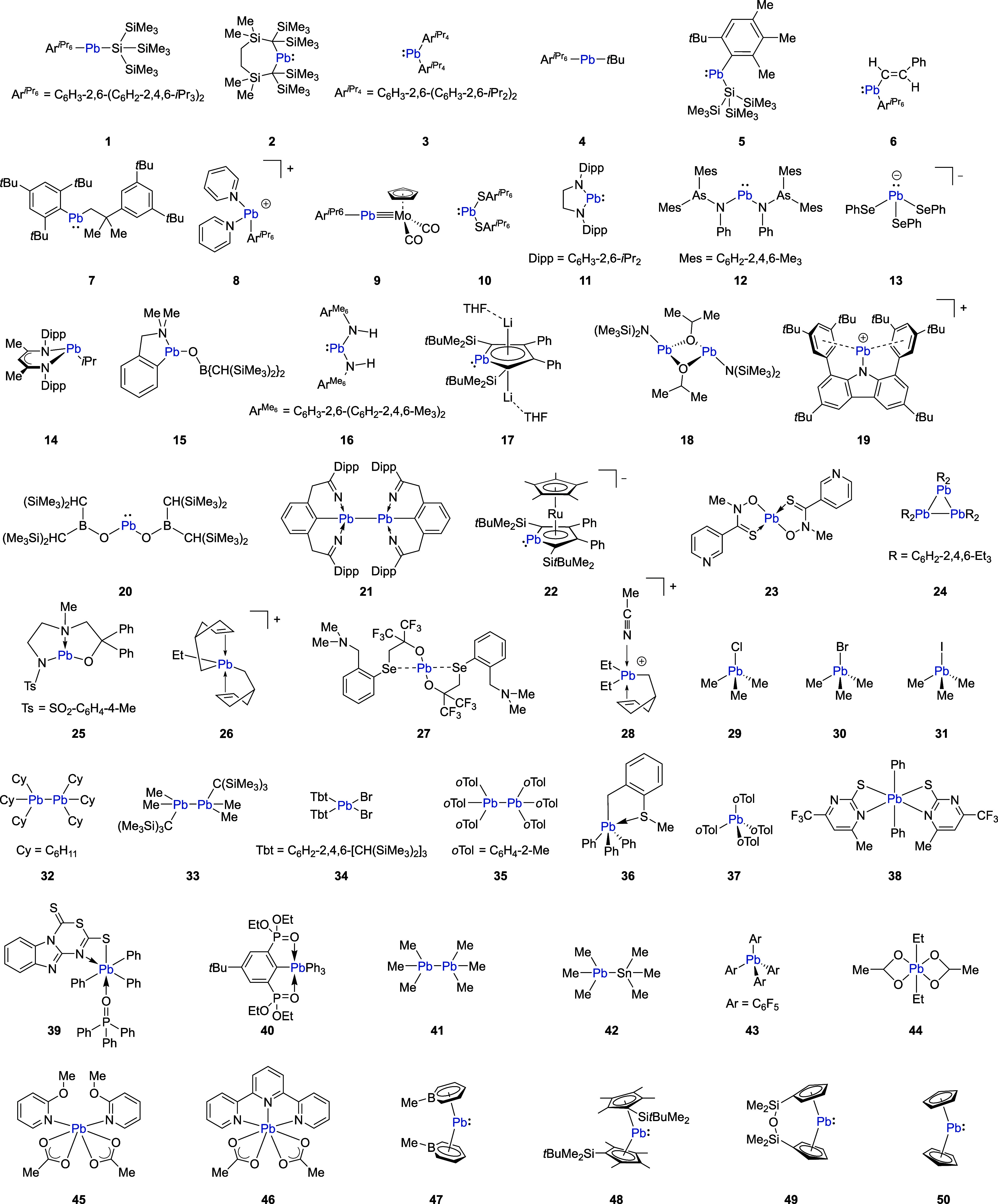
All compounds included in the *PbS50* benchmark study.

**Figure 2:**
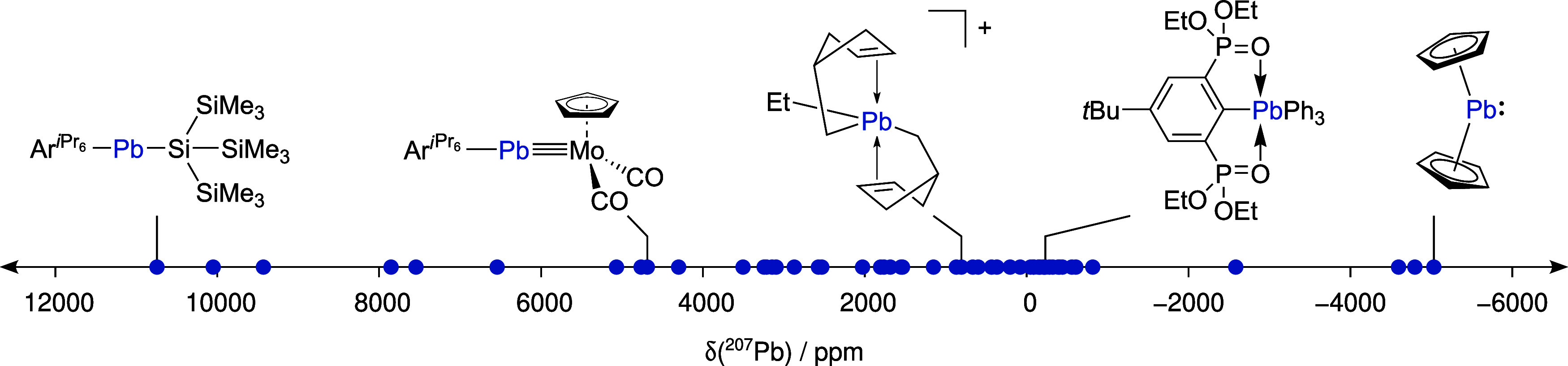
Overview of all experimentally determined ^207^Pb NMR chemical shifts included in this study.

The newly compiled *PbS50* benchmark set includes CEs for 50 organolead compounds of various sizes ranging from 14 to 187 atoms, with experimental reference data measured in solution for 50 ^207^Pb NMR chemical shifts between +10745 and −5030 ppm. In total, 282 conformers are included. Numerous bonding motifs, such as formal single, double, triple, aromatic, or dative bonds, with up to seven bonding partners on one Pb atom are represented. The NMR shifts were recorded in six solvents: benzene, chloroform, dichloromethane, DMSO, THF, and toluene. All structures are depicted in Figure 1, and an overview of all experimental ^207^Pb NMR chemical shifts is shown in Figure 2. The experimental shifts and solvents that were used in the NMR experiment are listed in Table 2. Additional details can be found in the Supporting Information.

**Table 2:** Experimental ^207^Pb NMR Chemical Shifts for All Compounds in the *PbS50* Set, the Solvents Used during the Measurement, and the Respective Reference

no	solvent	δ(^207^Pb)/ppm	refs
1	benzene	10 745	(92)
2	benzene	10 050	(93)
3	benzene	9430	(92)
4	benzene	7853	(92)
5	benzene	7545	(94)
6	benzene	6543	(95)
7	benzene	5067	(94)
8	benzene	4764	(96)
9	benzene	4686	(96)
10	benzene	4299.2	(97)
11	benzene	3504	(98)
12	benzene	3244	(99)
13	chloroform	3214	(100)
14	benzene	3145	(101)
15	benzene	3095	(102)
16	benzene	2871	(103)
17	benzene	2572.5	(104)
18	benzene	2531	(105)
19	dichloromethane	2027	(106)
20	benzene	1805	(102)
21	benzene	1684	(107)
22	benzene	1552.5	(108)
23	chloroform	1534	(109)
24	toluene	1152	(110)
25	DMSO	870	(111)
26	toluene	806.7	(112)
27	benzene	665	(113)
28	benzene	598	(114)
29	dichloromethane	432	(115)
30	dichloromethane	367	(115)
31	dichloromethane	203.6	(115)
32	benzene	80.2	(116)
33	benzene	–48.2	(117)
34	chloroform	–83	(118)
35	chloroform	–88.7	(119)
36	chloroform	–146.2	(120)
37	chloroform	–166.7	(119)
38	chloroform	–206.7	(121)
39	THF	–214.5	(122)
40	benzene	–231	(123)
41	benzene	–281	(124)
42	benzene	–324	(124)
43	chloroform	–391	(125)
44	chloroform	–441	(124)
45	chloroform	–558.9	(126)
46	DMSO	–816.68	(127)
47	dichloromethane	–2584	(128)
48	benzene	–4595	(129)
49	benzene	–4795	(130)
50	benzene	–5030	(131)

### Study of Density Functional Approximations and Relativistic Approaches for the Calculation of ^207^Pb NMR Chemical Shifts

**Figure 3:**
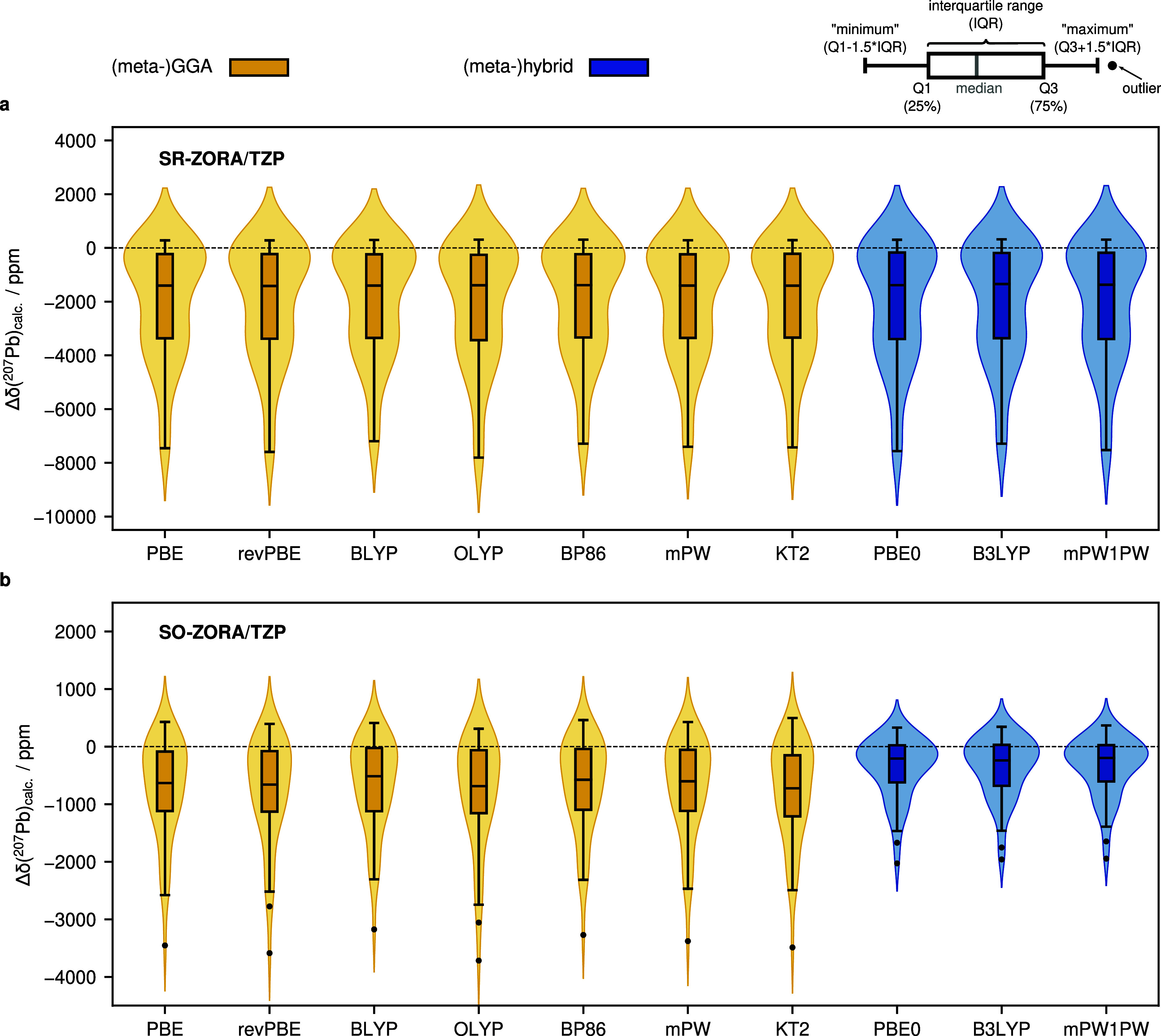
^207^Pb NMR shift deviation between calculation (ensemble-averaged shifts) and experiment represented in violin plots (Δδ = δ_calc_ – δ_exp_) for all tested DFAs using the SR (a) and SO relativistic (b) ZORA approach. The central lines represent the median values, the boxes the range of 25–75% of the data, and the whiskers all points within 1.5 times the interquartile range. Outliers are depicted by black dots.

The performance of different DFAs is assessed in combination with the ZORA/TZP basis set on the *PbS50* benchmark set for the computation of the ^207^Pb NMR chemical shifts. If not stated otherwise, the calculated NMR results refer to the ensemble-averaged NMR chemical shifts based on the Boltzmann weights at room temperature. A comparison of all tested DFAs is given in Figure 3, and the respective statistical data are listed in Table 3.

**Table 3:** Mean Deviation (MD), Mean Absolute Deviation (MAD), Standard Deviation (SD), and Root Mean Square Deviation (RMSD) of the Ensemble-Based Computed ^207^Pb NMR Chemical Shifts in ppm as Well as Their Respective Coefficient of Determination (*R*^2^)^a^

DFA	MD	MAD	SD	RMSD	*R*^2^	MAD_scaled_	MAD_lowconf_
SR-ZORA/ZORA/TZP							
PBE	–1996	2038	2131	2904	0.7211	1807	2038
revPBE	–2022	2062	2163	2945	0.7090	1858	2062
BLYP	–1950	1999	2076	2833	0.7356	1751	1999
OLYP	–2083	2123	2225	3032	0.6772	2002	2123
BP86	–1958	2005	2093	2850	0.7323	1761	2005
mPW	–1985	2029	2118	2887	0.7239	1797	2029
KT2	–1992	2033	2117	2891	0.7308	1762	2034
PBE0	–2018	2065	2205	2972	0.6808	2018	2066
B3LYP	–1970	2025	2140	2893	0.7022	1921	2025
mPW1PW	–2011	2060	2197	2962	0.6822	2013	2061
SO-ZORA/ZORA/TZP							
PBE	–731	801	863	1124	0.9605	519	805
revPBE	–756	823	895	1165	0.9580	537	827
BLYP	–668	748	807	1041	0.9632	510	751
OLYP	–787	856	941	1219	0.9516	582	860
BP86	–679	759	823	1061	0.9626	506	763
mPW	–707	780	844	1094	0.9615	513	784
KT2	–773	844	866	1154	0.9608	527	848
PBE0	–373	446	526	640	0.9828	342	449
B3LYP	–360	451	530	636	0.9811	364	457
mPW1PW	–350	429	510	614	0.9831	338	433

a Note that the mean deviation of the linear scaling approach is, by definition, always zero. Further, the MAD of the scaled (MAD_scaled_) and of the lowest conformer in each ensemble (MAD_lowconf_) are presented.

Relativistic effects have an immense influence on the ^207^Pb NMR chemical shift, which was also observed for the computation of solid-state ^207^Pb NMR shifts.^19,132^ The explicit treatment of only SR effects is insufficient to describe the chemical environment around the Pb nucleus (Figure 3a). The mean absolute deviations (MADs) range from 1999 to 2123 ppm, and the mean deviations (MDs) range from −1950 to −2083 ppm, indicating an underestimation for the majority of ^207^Pb NMR chemical shifts. The standard deviations are in a similar range as the MADs and therefore represent a large spread of errors. This results in small coefficients of determination, with the average over all methods being *R*_av._^2^ = 0.7095. In contrast to our previous work on ^29^Si and ^119^Sn NMR chemical shift computations, the accuracy of ^207^Pb NMR chemical shifts does not differ significantly with different density functionals when SR-ZORA is applied. The largest errors were obtained with OLYP (MAD = 2123 ppm) and the lowest errors with BYLP (MAD = 1999 ppm), but the difference is negligible considering the range of investigated shifts. Especially plumbylenes and some systems with heavy-atom effects on heavy-atom shielding (HAHA), such as **9**, are described poorly.

**Figure 4:**
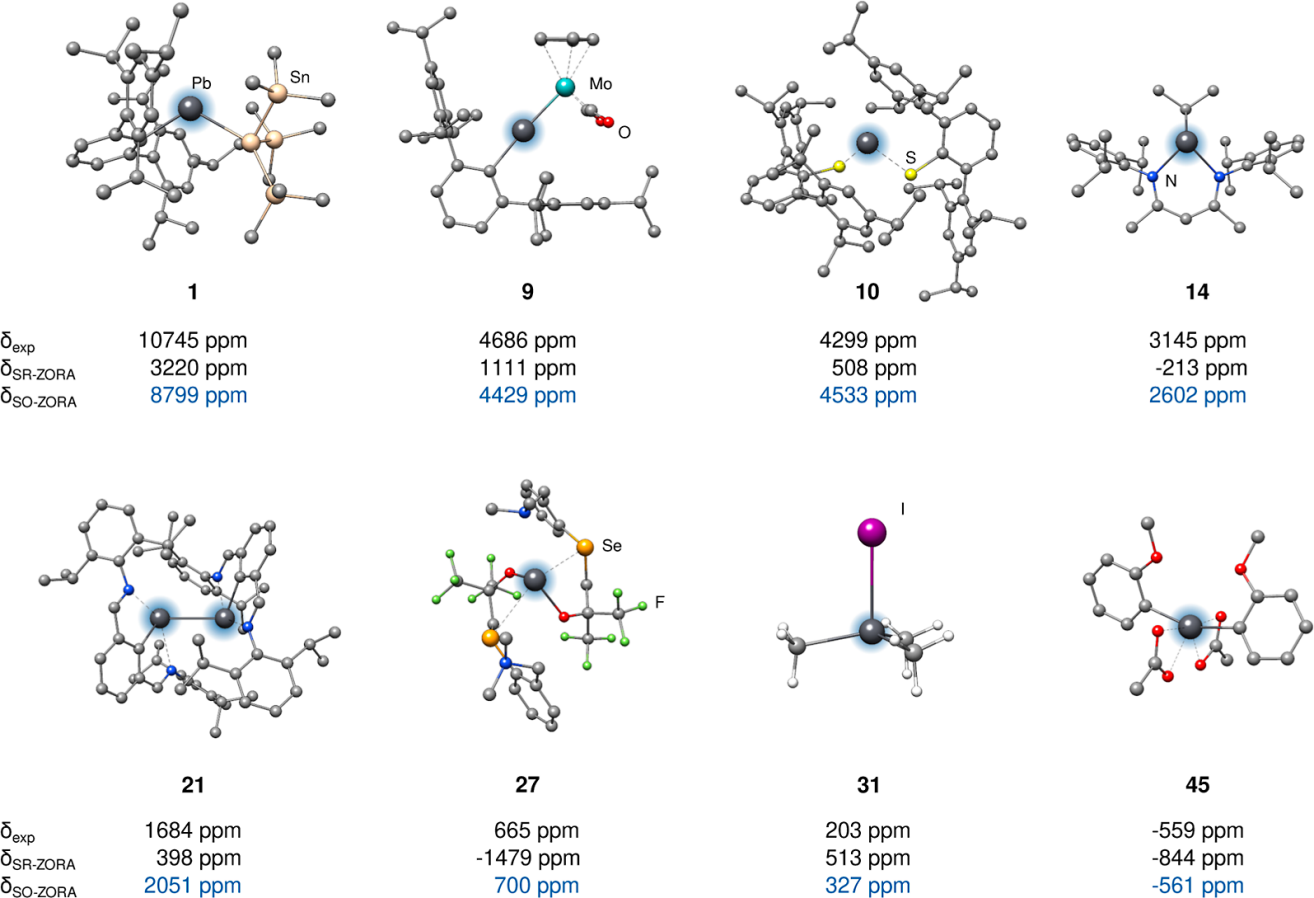
Selected systems of the *PbS50* set with their respective experimental and calculated ^207^Pb NMR chemical shifts computed at the mPW1PW/TZP level of theory. The most accurately calculated NMR shifts are highlighted in blue.

By incorporating SO coupling into the calculation, the results become considerably more accurate, as former outlier values are improved significantly. Selected examples are shown in Figure 4. The MADs lie between 856 and 429 ppm and are hence reduced by a factor of almost five. Hybrid functionals systematically outperform GGAs and yield lower errors. Therefore, Fock exchange is required for more reliable results, which is in line with the “Jacob’s Ladder” picture of DFAs and was also observed for the computation of solid-state ^207^Pb NMR shifts.^19,132^ In comparison to our ^29^Si and ^119^Sn NMR benchmark sets, smaller coefficients of determination are observed for ^207^Pb NMR chemical shifts. This underlines the challenging computation of heavier elements. Outliers are mainly compounds with positively charged Pb atoms and plumbylenes, which have complex electronic structures that are difficult to describe. This is also in line with our previous work, where large errors were observed for silylenes and stannylenes. The overall best-performing DFAs are PBE0 (MAD = 446 ppm, *R*^2^ = 0.9828) and mPW1PW (MAD = 429 ppm, *R*^2^ = 0.9831).

Based on the best-performing DFA, we selected a subset of the *PbS50* set where the SO contributions are below 200 ppm. This allows for a screening of a wider variety of density functionals, such as meta-GGAs or range-separated hybrids (RSHs) (see Supporting Information for further details). No significant improvement over the remaining calculations was observed, but the RSHs ωB97X-V^69^ and ωB97M-V^70^ yield a slightly lower MAD than the tested global hybrids. Therefore, RSHs should be investigated in future studies.

### Linear Scaling Correction

Errors inherent to the underlying theoretical method for the calculation of NMR chemical shifts are routinely corrected by an empirical linear scaling approach.^133−137^ In the following, the correction is applied to the ^207^Pb NMR chemical shifts according to
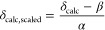
3with α being the slope and β being the intercept of the linear regression done for the calculated ^207^Pb NMR chemical shift with respect to the experimental reference data. The two parameters for each DFA can be found in the Supporting Information.

**Figure 5:**
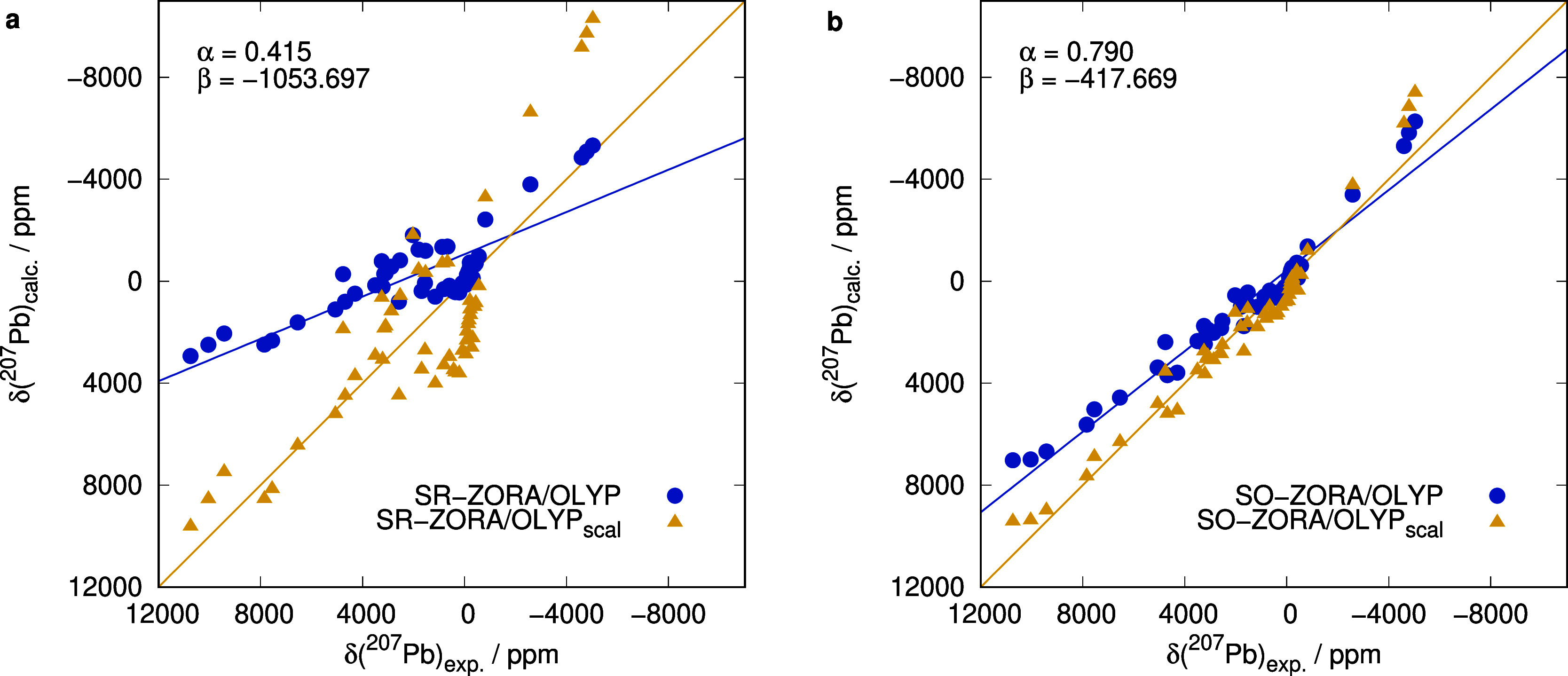
Correlation plots showing the calculated and experimental ^207^Pb NMR shifts for the worst performing DFA OLYP/TZP applying (a) SR-ZORA and (b) SO-ZORA. The plots also show a comparison between the unscaled and scaled (scal) results.

By scaling the calculated shifts, the mean deviation vanishes, and the MAD is decreased (Figure 5). The MAD is lowered from 1999 to 1751 ppm in the SR-ZORA approach for BLYP. Nevertheless, the error spread for SR-ZORA is still too large, and therefore, the corrected results are not reliable. If the SO-ZORA results are scaled, however, the MAD is also decreased going from 856 to 582 ppm, thus almost reaching the accuracy of the computationally more expensive hybrid DFAs. When the correction is applied to the results computed with hybrid functionals, the error is even further reduced, leading to the overall best performance. For example, the MAD of mPW1PW is reduced from 429 to 338 ppm and the MAD of PBE0 from 446 to 341 ppm. These results should still be taken with caution as a linear scaling correction might hide intrinsic errors in the calculations.

### Conformational Analysis

The impact of distinct conformers on the NMR chemical shift can be large in flexible structures.^138−140^ The results in the previous sections were obtained by averaging the calculated ^207^Pb NMR shift of each compound based on their Boltzmann weights, but in the majority of cases, the most populated conformer dominates the NMR chemical shift. Table 3 lists the MADs for the results obtained only for the lowest energy conformer (MAD_lowconf_). The MAD_lowconf_ is always slightly larger than the Boltzmann-averaged MAD, but the difference is 5.5 ppm at most and is therefore negligible. Since most structures in the test set are considered to be rigid and primarily contain only one central Pb atom, the conformational influence was expected to be small. The largest influence is observed for compound **19**, with δ_lowconf_ = 853 ppm and δ_ensemble_ = 986 ppm when calculated with SO-ZORA-PBE0/TZP. The corresponding experimental shift is δ_exp,19_ = 2027 ppm. The reason for the difference is the different coordination of phenyl rings in the structure. The lowest conformer features an η^6^ coordination of parallel-aligned phenyl rings to the central Pb atom. The less-populated conformers, however, exhibit differently coordinated phenyl rings that are not oriented parallel. Only the combination of both bonding motives leads to the most accurately computed ^207^Pb NMR chemical shift. This example illustrates the importance of conformational influences for a more accurate representation of the experimental NMR chemical shifts.

### Structure Dependence

**Figure 6:**
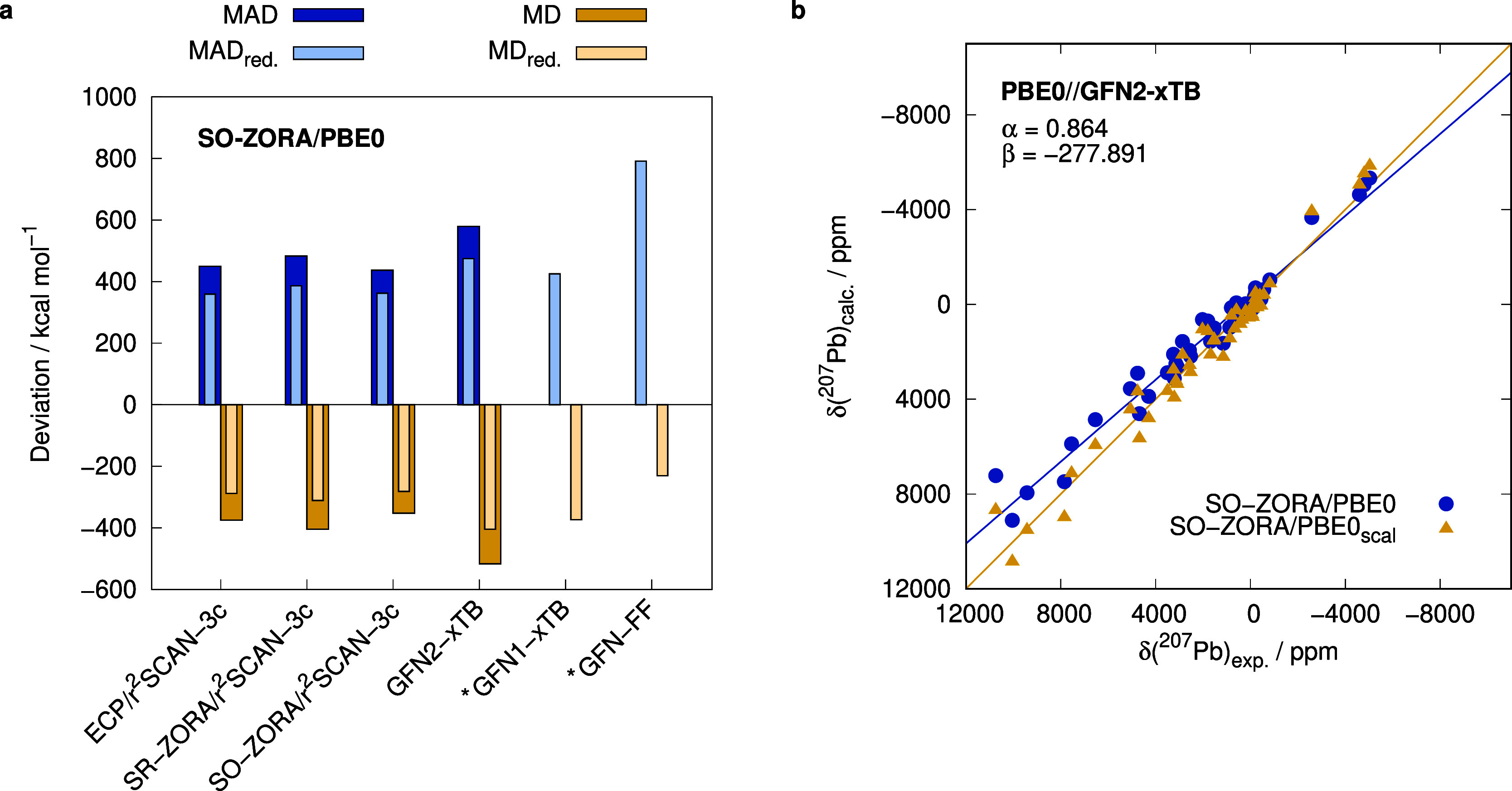
(a) MAD and MD of the ^207^Pb NMR shift calculated with SO-ZORA/PBE0/TZP for different levels of geometry optimization and (b) correlation plot between the calculated and experimental ^207^Pb NMR shift for GFN2-xTB geometries, including unscaled and scaled (scal) results. Plot (b) contains all 50 data points. *Compounds **1**, **2**, **3**, **5**, **9**, **19**, **21**, **33**, and **46** were removed from the test set to obtain the reduced mean (absolute) deviations MAD_red._ and MD_red._ as these systems did not converge or were poorly optimized by GFN1-xTB and GFN-FF.

The accuracy of the NMR chemical shift calculation depends on the quality of the equilibrium structure obtained by geometry optimization. Hence, different levels of theory for the optimization are evaluated in this section (Figure 6). The treatment of relativistic effects is compared for effective core potentials (ECPs), SR-, and SO-ZORA. In addition, the SQM methods GFN1-xTB, GFN2-xTB, and the general force field GFN-FF are investigated. For this, the geometry of the lowest conformer in each ensemble was reoptimized at the respective level of theory, and the ^207^Pb NMR chemical shifts were computed at the SO-ZORA/PBE0/TZP level of theory.

The treatment of relativistic effects is essential for reliable geometries that contain heavy elements such as Pb.^141^ These can be treated implicitly either by, e.g., ECPs that include relativistic reference data in their fit or by an all-electron treatment such as ZORA. The Stuttgart-Cologne def2-ECPs, which were applied for the geometry optimization of all conformers in the ensembles, yield very similar results as the computationally more demanding SO variant of ZORA in combination with the composite r^2^SCAN-3c DFT method. The respective MADs in subsequent ^207^Pb NMR chemical shift calculations are 449 and 437 ppm. Also, the mean deviation is similar with −375 and −352 ppm, respectively. In the SR-ZORA approach, where SO coupling is not considered, the errors are slightly larger. The MAD is 483 ppm, and the MD is −405 ppm. Since ECPs are more common and usually computed faster than all-electron approaches when several heavy nuclei are present, it is advised to apply them in the geometry optimization, for which many energy and gradient computations have to be performed.

Even more efficient geometry optimizations are possible with semiempirical or force field methods. However, some calculations with the GFN1-xTB and GFN-FF methods do not converge for all systems or yield qualitatively wrong geometries. If these compounds are removed from the test set, the workflow still yields reasonable results. The MADs of the reduced test set (MAD_red._) are 425 and 792 ppm for GFN1-xTB and GFN-FF, respectively. In comparison, the ECP-based r^2^SCAN-3c method yield an MAD_red._ of 359 ppm.

The GFN2-xTB method has an MAD_red._ of 474 ppm, which is slightly worse than that of GFN1-xTB, but it is more robust as all geometries were optimized correctly. The results can further be improved by applying the aforementioned linear scaling approach (Figure 6b), which here mainly corrects for systematically incorrect bond lengths. After linearly scaling the NMR chemical shifts, an MAD_scaled_ of 409 ppm is achieved, which is lower than the unscaled results obtained from r^2^SCAN-3c/ECP geometries (MAD = 449 ppm). Therefore, geometries obtained with GFN2-xTB represent a viable alternative for the computation of ^207^Pb NMR chemical shifts. As the computations are much faster than DFT optimizations, this can, e.g., be applied in large-scale screening processes.

## Conclusions

The computation of NMR chemical shifts can facilitate experimental measurements and support the evaluation of measured spectra. In this work, a comprehensive benchmark set for the computation of ^207^Pb NMR chemical shifts was introduced, which features 50 experimental ^207^Pb NMR chemical shifts measured in solution. It includes conformers of 50 compounds with various bonding patterns at the lead center. The set is aimed to be used for the evaluation of NMR prediction methods, but it can also be regarded as a database to find structures that exhibit ^207^Pb NMR signals in a similar range. Herein, it was used to evaluate the performance of DFT and the influence of conformational and relativistic effects in the computation of the ^207^Pb NMR chemical shifts. Further, the effect of the underlying level on geometry optimization was assessed.

Relativistic effects were explicitly treated by SR- and SO-ZORA in the calculations. When SR-ZORA is applied in the ^207^Pb NMR shift calculation, the shifts are on average underestimated, and the errors are immense, regardless of the used DFA. By applying SO-ZORA, the results are greatly improved, as expected, for the heavy element Pb. Inclusion of SO effects for the calculation of NMR chemical shifts is not very common in quantum chemical codes, yet. Therefore, efforts for additive corrections, e.g., by applying machine learning approaches, have already been made.^142^ Hybrid density functionals were found to generally outperform the GGA functionals.

As the majority of tested systems are rigid, conformational effects are small. For most cases, it suffices to compute the NMR chemical shift only for the most stable conformer. Nevertheless, special cases require the inclusion of some low-lying conformers in order to predict the correct NMR shifts. For example, the ^207^Pb NMR chemical shift of compound **19** is computed more accurately, and the error is reduced by over 130 ppm when the shift is ensemble-averaged.

Our test on the level of theory applied in the geometry optimization showed that the explicit treatment of relativistic effects is negligible, and ECPs should be used instead for substantial computational time savings. Faster geometry optimizations are possible with the semiempirical GFN2-xTB method. The errors in a subsequent ^207^Pb NMR chemical shift calculation are still reasonable if the shifts are scaled linearly. Therefore, this approach can be successfully used in screening processes.

The overall best-performing method combinations for the computation of ^207^Pb NMR chemical shifts evaluated on the *PbS50* set are PBE0/ZORA/TZP (MAD = 446 ppm) and mPW1PW/ZORA/TZP (MAD = 429 ppm) in combination with the SO variant of ZORA.

## Supporting Information Available

Geometry files for each compound of the PbS50 set are in XYZ format (pbs50.zip) (ZIP)
Computed ^207^Pb NMR shielding and shift values (XLSX)
More detailed computational settings and supporting figures (PDF)
