# Supplementary material for: Benchmark Study on the Calculation of 207Pb NMR Chemical Shifts
Source: Inorg Chem. 2024 Mar 6;63(11):5052–64. doi: 10.1021/acs.inorgchem.3c04539 (PMC10951955; doi:10.1021/acs.inorgchem.3c04539)
Supplement: Supplementary file 3 — ic3c04539_si_003.pdf [file ic3c04539_si_003.pdf]

Supporting Information

Benchmark Study on the Calculation of ^{207}Pb NMR Chemical Shifts

Thomas Gasevic,† Julius B. Kleine Büning,† Stefan Grimme†*, and Markus Bursch‡*

†*Mulliken Center for Theoretical Chemistry, Clausius Institute for Physical and Theoretical Chemistry, University of Bonn, Beringstr. 4, 53115 Bonn, Germany*

‡*Max-Planck-Institut für Kohlenforschung, Kaiser-Wilhelm-Platz 1, 45470 Mülheim an der Ruhr, Germany*

bursch@kofo.mpg.de

grimme@thch.uni-bonn.de

Contents

1 Computational Details **S3**

1.1 General . . . S3

1.2 SR-ZORA Subset . . . S4

2 Statistical Quantities **S4**

3 Additional Figures **S5**

3.1 Linear Scaling Approach . . . S5

3.2 Geometry Study . . . S5

3.3 NMR Shift Components . . . S5

1 Computational Details

1.1 General

Table S1: Dielectric constants ε and solvent molecule radii in Å that were applied for the COSMO solvation model in the calculations.

Solvent	ε	Radius / Å
Benzene	2.28	3.28
Chloroform	4.81	3.17
Dichloromethane	8.93	2.94
Dimethylsulfoxide	46.7	3.04
Tetrahydrofuran	7.58	3.18
Toluene	2.38	3.48

For each structure contained in the presented study, the final geometry was optimized with r^{2}SCAN-3c/mTZVPP and the COSMO implicit solvation model. The free energy contribution G_{mRRHO} was obtained via the modified rigid-rotor harmonic-oscillator (mR-RHO) approximation based on the evaluation of vibrational frequencies on the GFN2-xTB level of theory. The solvation free energies δG_{solv} were calculated with COSMO-RS at 298.15 K and the BP_TZVP_C30_1601 parametrization. The total free energy of a compound G_{c}, which is used for the conformer ranking and the determination of the Boltzmann weights, is the sum of the total r^{2}SCAN-3c single-point energy E_{c} and the free energy contributions (eq (1)).

$$G_{\mathrm{c}} = E_{\mathrm{c}} + G_{\mathrm{mRRHO}} + \delta G_{\mathrm{solv}} \tag{1}$$

Further calculation settings of the conformer searches and NMR shift calculations are listed in Tables S2 and S3.

Table S2: Calculation settings for the ensemble generation.

Program	Task		Theory level	Solvation	Sorting threshold
CREST	conf. search		GFN-FF or GFN2-xTB	ALPB	6.0 kcal mol^{-1}
CENSO	ensemble	part 0	B97-D3 // CREST structures	ALPB	6.0 kcal mol^{-1}
	refinement	part 1	r^{2}SCAN-3c // CREST structures	COSMO-RS	5.5 kcal mol^{-1}
		part 2	r^{2}SCAN-3c // r^{2}SCAN-3c	COSMO, COSMO-RS	2.5 kcal mol^{-1}

Table S3: Calculation settings for subsequent geometry optimizations and calculation of NMR parameters.

Program	Task	Grid	RI	Auxbasis	Solvation	Miscellaneous
TURBOMOLE	geo. opt.	m5	RIJ	def2/J	COMSO	`$symmetry c1`
	NMR	5a	RIJ	default	COMSO	`$symmetry c1`
AMS/ADF	geo. opt., NMR	`NumericalQuality good`	default	default	COSMO, `Surf Esurf`, `div ndiv=5`	`Symmetry nosym`

1.2 SR-ZORA Subset

For a subset consisting of compounds **26**, **28-43**, and **48** the spin-orbit contribution was found to be below 200 ppm. All results of the evaluated density functionals can be found in the ESI.xlsx file.

2 Statistical Quantities

The following quantities were used for the statistical evaluation of the chemical shift data of the benchmark studies with n data points. δ_x denotes the calculated and δ_r the reference, i.e. the experimental value.

Mean deviation (MD):

$$MD = \frac{1}{n}\sum_{i}^{n}(\delta_{x_i} - \delta_{r_i}) \tag{2}$$

Mean absolute deviation (MAD):

$$MAD = \frac{1}{n}\sum_{i}^{n}(|\delta_{x_i} - \delta_{r_i}|) \tag{3}$$

Root mean square deviation (RMSD):

$$RMSD = \sqrt{\frac{1}{n}\sum_{i}^{n}\left(\delta_{x_i} - \delta_{r_i}\right)^2} \tag{4}$$

Determination coefficient (R^2):

$$R^2 = 1 - \frac{\sum_i^n (\delta_{x_i} - \delta_{r_i})^2}{\sum_i^n (\delta_{x_i} - \bar{\delta}_x)^2} \tag{5}$$

All calculated and experimental values can be found in the ESI.xlsx file.

3 Additional Figures

3.1 Linear Scaling Approach

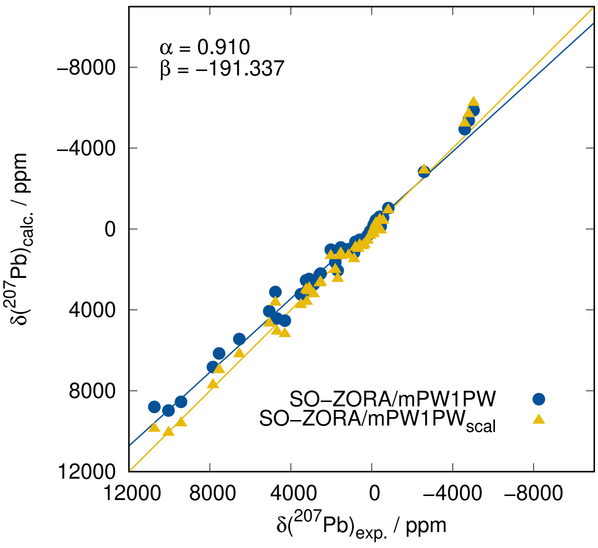

Figure S1: Correlation plot showing the calculated and the experimental ^{207}Pb NMR chemical shifts for the best performing DFA mPW1PW/TZP with SO-ZORA. The plot also shows a comparison between the unscaled and linearly scaled (scal) results.

3.2 Geometry Study

3.3 NMR Shift Components

The ^{207}Pb NMR chemical shielding highly depends on the paramagnetic contribution and is therefore sensitive to the chemical environment (Figure S3). We do not observe any general effects originating from the oxidation state but a similar ligand sphere leads to similar paramagnetic shieldings (see, for example, compounds **47 - 50**).

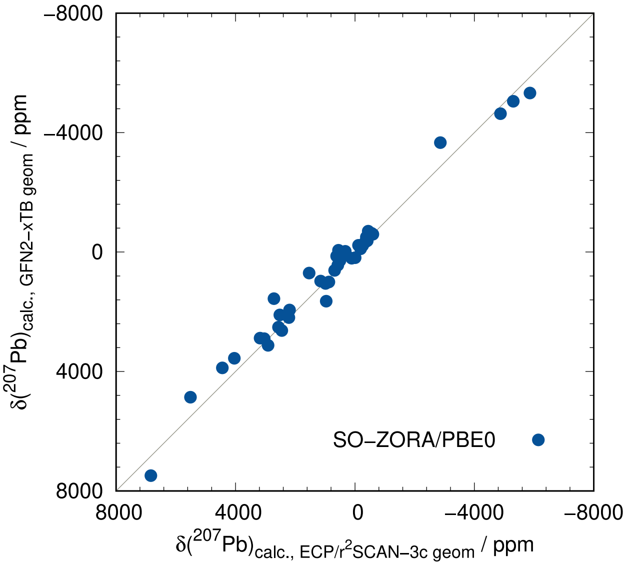

Figure S2: Correlation plot showing the correlation between ^{207}Pb NMR chemical shifts computed with geometries optimized with ECP/r^{2}SCAN-3c and GFN2-xTB.

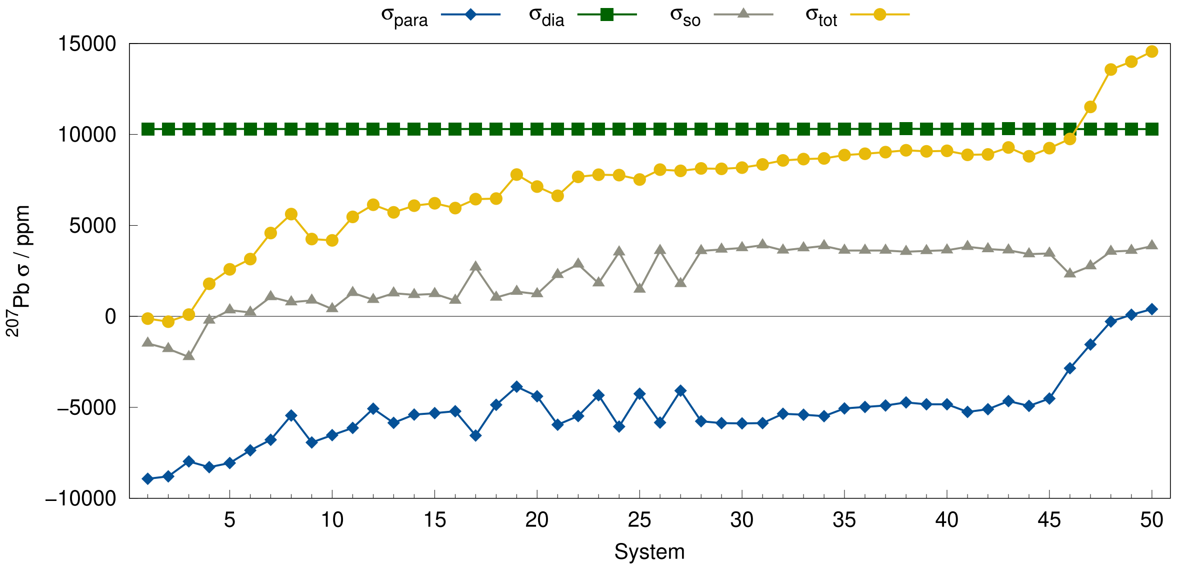

Figure S3: Paramagnetic (σ_{para}), diamagnetic (σ_{dia}) and spin-orbit (σ_{so}) contributions to the total ^{207}Pb NMR chemical shielding (σ_{tot}) computed with mPW1PW/TZP and SO-ZORA on the lowest conformer of each compound.
